# Comparison of a percutaneous device and the bougie-assisted surgical technique for emergency cricothyrotomy: an experimental study on a porcine model performed by air ambulance anaesthesiologists

**DOI:** 10.1186/1757-7241-21-59

**Published:** 2013-07-26

**Authors:** Anders R Nakstad, Per P Bredmose, Mårten Sandberg

**Affiliations:** 1 Air Ambulance Department, Division of Emergencies and Critical Care, Oslo University Hospital, Sykehusveien 19, N-1474 Nordbyhagen, Norway; 2 University of Oslo, Oslo, Norway

**Keywords:** Emergency cricothyrotomy, Cannot intubate -cannot ventilate, Pre-hospital airway management

## Abstract

**Background:**

A large number of techniques and devices for cricothyroidotomy have been developed. In this study, the Portex™ Cricothyroidotomy Kit (PCK, Smiths Medical Ltd, Hythe, UK) was compared with the bougie assisted emergency surgical cricothyrotomy technique (BACT).

**Methods:**

Twenty air ambulance anaesthesiologists performed emergency cricothyrotomy on a cadaveric porcine airway model using both PCK and BACT. Baseline performance and performance after the intensive training package were recorded. Success rate, time to secured airway and tracheal damage were the primary endpoints, and confidence rating was a secondary endpoint.

**Results:**

During baseline testing, success rates for PCK and BACT were 60% and 95%, respectively. Tracheal injury rate with PCK was 60% while no such injury was found in BACT. A lecture was given and skills were trained until the participants were able to perform five consecutive successful procedures with both techniques. In the post-training test, all participants were successful with either technique. The mean time to successful insertion was reduced by 15.7 seconds (from 36.3 seconds to 20.6 seconds, p< 0.001) for PCK and by 7.8 seconds (from 44.9 seconds to 37.1 seconds, p=0.021) for BACT. In the post-training scenario, securing the airway with PCK was significantly faster than with BACT (p<0.001). Post-training tracheal laceration occurred in six (30%) of the PCK procedures and in none of the BACT procedures (p=0.028).

The self-evaluated confidence level was measured both pre- and post-training using a confidence scale with 10 indicating maximum amount of confidence. The median values increased from 4 to 8 for PCK and from 6.5 to 9.5 for BACT. All participants reported that BACT was their preferred technique.

**Conclusions:**

Testing the base-line PCK skills of prehospital anaesthesiologists revealed low confidence, sub-optimal performance and a very high failure rate. The BACT technique demonstrated a significantly higher success rate and no tracheal damage. In spite of PCK being a significantly faster technique in the post-training test, the anaesthesiologists still reported a higher confidence in BACT. Limitations of the cadaveric porcine airway may have influenced this study because the airway did not challenge the clinicians with realistic tissue bleeding.

## Background

A number of critically ill or injured patients need immediate airway management including endotracheal intubation in the field. The reported need for emergency cricothyrotomy varies but is generally below one percent when prehospital intubation attempts are made by anaesthesiologists and experienced emergency physicians [1-5]. In two reports from services staffed with surgeons or flight nurses, a frequency above 10% is reported [6,7].

Independently of the medical system, occasionally a “cannot intubate – cannot ventilate” (CICV) situation will occur. In such instances, most airway management guidelines recommend that an emergency cricothyrotomy should be performed [8-10]. A number of techniques and devices have been developed to simplify the procedure. A recent systematic review is inconclusive with respect to the superiority of any one of the techniques [11].

The classic emergency surgical airway and the refined “rapid four step technique” (RFST) are both performed with an incision through the cricothyroid membrane and the use of a tracheal hook and/or dilatators to secure the airway before a tube is advanced into the tracheal lumen [12,13]. The “bougie-assisted emergency cricothyrotomy” (BACT) described by Hill and co-workers is the most recent refinement [14]. This technique makes use of simple equipment including the bougie that may be used in earlier phases of the difficult airway algorithm. In BACT, a single stab incision is made through the cricothyroid membrane and a bougie is used to secure the access before an endotracheal tube is inserted into the tracheal lumen. A tracheal hook is angled caudally and – if necessary – a Trousseau dilatator is used to secure access to the tracheal lumen.

The commercial kits for emergency cricothyrotomy can be divided into two broad categories. One group consists of kits based on the Seldinger technique that punctures the cricothyroid membrane followed by the insertion of a guide wire through the needle. These kits include the Arndt emergency cricothyrotomy catheter set (Cook, Bloomington, IL, USA), the Melker wire-guided cricothyrotomy set (Cook, Bloomington, IL, USA) and the Minitrach II (Smiths Medical Ltd, Hythe, UK) [15-18]. The other group of commercial kits are based on a cutting device that creates a lumen in the cricothyroid membrane that is sufficiently wide for the insertion of the endotracheal tube included in the kit. Examples of such products are the QuickTrach2 kit (VBM Medizintechnik GmbH, Sulz, Germany) and the Portex cricothyrotomy kit (Smiths Medical Ltd, Hythe, UK) [19-21].

The Portex cricothyrotomy kit (PCK) has been a part of airway management equipment in several Norwegian Emergency Medical Services (EMS) in recent years [21]. Despite the availability of the device, an evaluation of the activity of the Oslo University Hospital Helicopter Emergency Medical Service (HEMS) revealed that when an emergency surgical airway was needed, the anaesthesiologists appeared not to prefer the PCK but instead used a modified RFST technique.

With the large number of available techniques and devices for emergency prehospital cricothyrotomy and an infrequent need of the intervention in real life, it is of great interest to evaluate the most promising methods. Thus, the aim of this study was to evaluate the performance of the two locally available techniques when performed by air ambulance anaesthesiologists in both a baseline setting and after an intensive training package.

## Methods

### Study design

This was a prospective, randomised, crossover trial comparing PCK and BACT in a porcine airway model. Twenty air ambulance anaesthesiologists with a median post-graduate experience of 15 years (range 8–29) participated. None of the participants had extensive experience with either technique. All participants, however, had performed emergency cricothyrotomy in a porcine model with PCK and BACT during skill training one year prior to the present study. Six of the participants had previously performed an emergency cricothyrotomy in a real CICV-setting.

The protocol was presented to the regional medical ethics committee, who stated that the study did not need the committee's approval. All physicians participated voluntarily, and no data were attached to their identities.

### Cricothyrotomy techniques

When performing PCK, the larynx is immobilised and a vertical skin incision is made with a scalpel. Identification of the cricothyroid space is made by palpation before the locator spring-loaded needle is inserted perpendicular to the skin and pushed towards the membrane. Once the tracheal lumen is reached, the indicator flag disappears. The flag reappears when the tip of the device makes contact with the posterior tracheal wall. At this point, the device is angled caudally, the flag once again disappears and the device is inserted another 1–2 cm before the needle is removed. The cricothyrotomy tube is slid over the dilatator into the tracheal lumen before the dilatator is removed.

In BACT, the provider is located lateral to the patient and stabilises the trachea with the thumb and the middle finger of the non-dominant hand while identifying the cricothyroid membrane with the index finger of the dominant hand. When identified, a transverse stabbing incision is made through the membrane with a scalpel. A tracheal hook is applied at the caudal margin of the incision and the hook is pulled caudally. A bougie is inserted through the incision into the trachea, and a 6.0 mm endotracheal tube is inserted over the bougie into the tracheal lumen before the bougie is removed.

### Anatomic model

**Figure 1:**
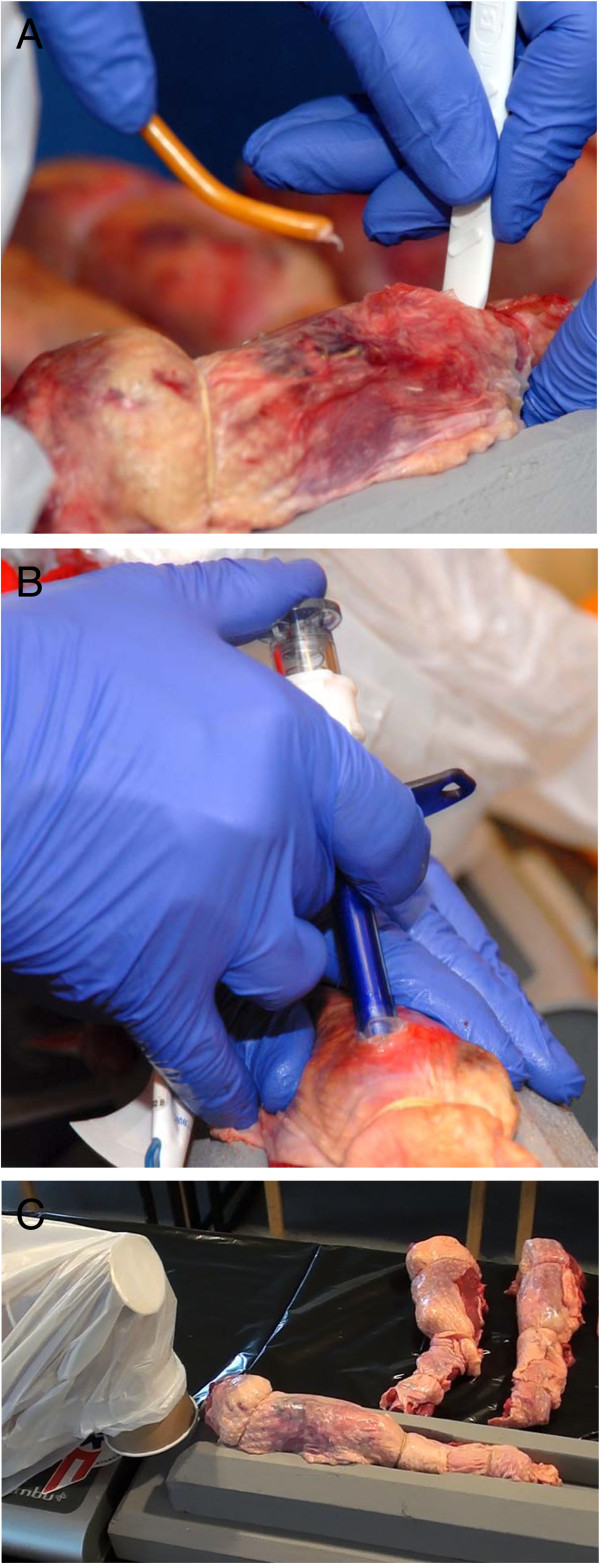
**Simulated emergency cricothyrotomy in a cadaveric porcine model. A)** The picture shows the perforation of the cricothyroid membrane in the BACT technique performed in the porcine airway model. **B)** The picture shows the perforation of the cricothyroid membrane with the PCK device in the porcine airway model. **C)** The porcine larynx is attached to a device and cranial space is limited by the prescience of the head of an airway simulator that is modified and orientated to mimic the anatomy of an adult male patient with flexion of the neck.

A model based on larynxes from adult pigs has been shown to be feasible for skill training in cricothyrotomy and for evaluation of new techniques [22,23]. The porcine larynx was fixed on a stand and covered with three layers of chicken skin to achieve realistic anatomy with respect to landmarks and sliding layers of tissue. Access to the larynx was restricted due to a manikin head located in an anatomically correct position cranially of the laryngeal model (Figure 1). Before each test, the larynx was inspected to ensure that there was no damage to the anatomical structures. The porcine larynx was replaced after each procedure and examined for grading of the tracheal wall lacerations and damage to the cartilage.

### Study protocol

Each participant was tested before and after training. In both instances, the order in which the two techniques were performed was randomised with a computer program. The first test evaluated the baseline skills with limited pre-test information. The post-training test was made at least six months later. The participants watched a 15-minute tutorial and participated in supervised hands-on training. All participants then performed between two and six procedures with the same porcine airway while given step by step verbal instructions and, if needed, demonstrations. When the technical sequence was performed correctly, a new porcine airway was prepared for each further consecutive attempt. These were made without interference by the instructor, but with verbal feedback. The participant continued until the cricothyrotomy performance was considered to have reached an adequate level, which was defined as five consecutive successful procedures. Use of more than 120 seconds to perform a correct procedure or misplacement of the endotracheal tube was set as the criteria for a non-successful attempt and in such cases the time would not be included in the data analysis.

The success rates, time to completion of the procedure and damage to the posterior tracheal wall were the primary outcomes. Time was recorded from the verbal command to start the procedure and continued until the tube/device was placed in the trachea, cuffed and ready to be connected to a self-inflatable bag. Whether the tracheal opening was secured by the tracheal hook or the dilatator during the BACT procedure was recorded, as well as whether the PCK was used in accordance with the written instructions of the product. Damage to the posterior wall of the trachea was graded using the posterior wall trauma score introduced by Murphy and co-workers with a grade of 0 for no laceration, a grade of 1 for minor (<5 mm) partial thickness laceration, a grade of 2 for major (>5 mm) partial thickness laceration and a grade of 3 for full thickness laceration [23].

The participants rated their confidence by responding to the question “How confident would you be in using this technique in a real CICV situation, graded 1–10, with 1 indicating no confidence and 10 indicating the maximum confidence that you believe is realistic in such a situation?” The question was given prior to and after both baseline and post-training tests.

### Data analysis

Data were analysed using Excel 2010 (Microsoft, Redmond, WA, USA), PASW Statistics version 18 (SPSS Inc. Chicago, IL, USA) and GraphPad InStat version 3.00 (GraphPad Software, San Diego, CA, USA). Fisher’s exact test was used for comparing frequencies, Unpaired T-test was employed for parametric data and the Kruskall-Wallis test with Dunn was employed for non-parametric data.

Parametric data are presented as the means with standard deviation (SD), whereas non-parametric continuous data are presented as the medians with inter-quartile range (IQR). Ordinal data and categorical data are presented as number and frequencies.

## Results

### Success rates, use of accessories and complications

Data describing success rates, use of accessory equipment and rate and type of tracheal damage are listed in Table 1.

**Table 1:** Success rates, use of accessory equipment and tracheal damage with PCK and BACT in baseline and post-training testing

**Characteristics**	**Baseline**	**Post-training**
**BACT**		
Success rate, n (%)	19 (95)	20 (100)
Use of dilatator, n (%)	10 (50) *	4 (20) *
Correct use of tracheal hook, n (%)	6 (30)	19 (95)
Use of both dilatator and hook, n (%)	3 (15)	4 (20)
Use of gum-elastic bougie, n (%)	20 (100)	20 (100)
Posterior wall trauma score, median (IQR)	0 (0)	0 (0)
**PCK**		
Success rate, n (%)	12 (60)	20 (100)
Laceration of posterior tracheal wall, n (%)	12 (60)	6 (30)
Use of scalpel, n (%)	10 (50)	3 (15)
Posterior wall trauma score, median (IQR)	2 (1–3)	1 (0–1)
Correct retraction of the locator needle, n (%)	3 (15) #	19 (95) #

p-values (when significant): * p = 0.04.

# p = 0.006.

In the baseline test, no unsuccessful attempts with any technique were due to excessive time to successful insertion. The single failure in performing BACT was due to the destruction of the cartilage and subsequent misplacement of the tube. The eight PCK failures were all due to placement of the tube in a false lumen caused by laceration of the posterior wall of the trachea or perforation through the tracheal wall.

In the post-training test, all participants were successful with both techniques. Post-training tracheal laceration occurred in six (30%) of the PCK procedures and in none of BACT procedures (p=0.028).

### Time to successful insertion

Data describing relevant time variables in both the baseline and post-training tests are listed in Table 2. The mean time to successful insertion was reduced by 15.7 seconds (from 36.3 seconds to 20.6 seconds, p< 0.001) for PCK and by 7.8 seconds (from 44.9 seconds to 37.1 seconds, p=0.021) for BACT. In the post-training scenario, PCK was significantly faster in securing the airway than BACT (p<0.001).

**Table 2:** Relevant time variables in baseline and post-training testing

	**Baseline**			**Post-training**		
	**Time from start of procedure (seconds)**			**Time from start of procedure (seconds)**		
	**Mean**	**Median**	**SD**	**Mean**	**Median**	**SD**
**BACT**						
Incision of membrane	7.8	7	3.0	8.4	6	4.6
Bougie in trachea	26.8	24	8.3	23.7	21	8.7
Tube fully advanced	39.7	39	9.4	30.8	27.5	11.2
Cuffed and ready to connect to the self-inflatable bag	44.9 *	45	9.8	37.1 * ^	33	8.8
**PCK**						
Perforation of the membrane	14.6	13	8.1	9.0	8	4.0
Tube advanced fully	30.9	29	11.8	15.2	14	5.4
Cuffed and ready to connect to the self-inflatable bag	36.3 ¤	33	10.4	20.6 ¤ ^	20.5	5.4

p-values (when significant): * p = 0.021.

¤ p < 0.001.

^ p < 0.001.

### Confidence rating

The median values for the self-evaluated confidence level at baseline and post-training tests increased from 4 to 8 for PCK and from 6.5 to 9.5 for BACT.

When asked directly before each test and after each test, all participants answered that BACT was their preferred technique if they were able to choose between them in a clinical situation.

## Discussion

### Baseline performance with PCK

The PCK baseline test resulted in a success rate of only 60%. All failures were due to placement of the tube in a false lumen or through the posterior tracheal wall. The success rate and laceration rate of the posterior tracheal wall was comparable to data from other studies in which inexperienced participants performed the procedure [23,24]. Small linear lacerations may be of no clinical importance while major lacerations with penetration through the tracheal wall may be catastrophic. The incidence of tracheal lacerations and misplacement of the tube may be a consequence of the construction of the device, but there are some alternative explanations. One important reason for the high frequency of posterior wall damage may be the delayed retraction of the needle by the user. In our study, only three participants retracted the steel needle at the correct point of time, and none of them caused any tracheal damage. Another reason may be the rigidity of tissue in this specific model, but this is less likely because posterior tracheal damage has been identified in different models.

In our study, the successful PCK attempts in the baseline test were performed with a time to successful insertion of median 33 seconds. This is similar to the results in the study by Assmann and co-workers were PCK was used in a fixed airway model [21]. The time to successful insertion in our study, however, is markedly less than the time to successful insertion of median 82 seconds reported in a study by Helm and co-workers where first year anaesthesia residents used PCK in a human cadaveric model [23]. It is also in contrast with the only report of use of PCK *in vivo* that we have identified in which time to successful insertion was 84 and 110 seconds in the two procedures that were reported [25]. The time to successful insertion using PCK in our study may be influenced both by our design and by the anatomic model. Participants were standing ready next to the model with all equipment available on a table when time recording started. The recording was stopped when the participant said “finished” and the tube was in place and cuffed but prior to the connection of a self-inflatable bag. A realistic preparation of equipment would likely increase the measured time to successful insertion. A lack of bleeding and a thinner soft tissue than in real patients may also influence the time to successful insertion in this study. Another factor may be the general clinical experience of the participants.

### Baseline performance with BACT

The BACT technique was more successful than PCK in the unprepared baseline setting and had a 95% success rate. However, detailed analysis of performance revealed that few participants performed the technique correctly. For instance, only six participants used the tracheal hook for its intended purpose. It was noted, however, that all participants continuously secured the opening into the trachea during both tests, and all participants used the gum-elastic bougie to further secure the airway before advancing the endotracheal tube into the trachea.

### Performance after the intensive training package

Post-training, all anaesthesiologists performed both techniques with a 100% success rate. The mean time to successful insertion was reduced by 17% for BACT and by 43% for PCK which may be of clinical importance. The marked improvement using PCK indicates that this technique may be performed incorrectly if the training of the providers is inadequate. After training, the PCK still generated some tracheal lacerations (n=12 versus n=6, p=0.27) but all were less than 5 mm in length and were superficial (Posterior Wall Trauma Score 1).

The success rates with BACT in this study are comparable to the study by Hill and co-workers, but the time to successful insertion (median 44 seconds in baseline and 37 seconds in post-training tests) were less than those reported by Hill (median 67 seconds) [14]. One explanation for this may be that different models were used. Hill and co-workers used anaesthetised sheep with a real bleeding risk and with subcutaneous tissue covering the trachea. The defined start and end-points were similar. Another factor that may explain the lower time to successful insertion in the present study may be the clinical experience of the participants.

The reduction in time to successful insertion for BACT was small, which may indicate that BACT is an intuitively easier technique that is not influenced by training to the same extent as PCK.

### Confidence and implications for choice of technique

The confidence in BACT may be seen as remarkable if one considers only the time to successful insertion and success rates found after the intensive training package. The participants, however, make up their mind based on previous experiences that include high failure rates in skill training a year prior to the study. In addition, the clinicians may evaluate the porcine airway model as artificial and that it is easier to perform a cricothyrotomy in this model than in a real clinical setting. Another explanation may be that the BACT is a simple technique with visual control while PCK is experienced as more difficult to perform.

The present study is one of few to compare base-line performance with optimised performance for two different but comparable techniques. We believe that the results add to the knowledge needed to make decisions regarding which difficult airway management strategy should be employed in this type of emergency medical service.

### Limitations of the study

A main limitation of this study was the use of a cadaveric model with no risk of bleeding and less soft tissue covering the airway structures than would be the case in a real human scenario. It is likely that this makes performance less difficult than would be the case in a real scenario. Despite this limitation, ethical and practical reasons made this model (using tissue from a porcine airway) the most suitable for our study.

Technical limitations and the anticipated training effect using the same type of model each time are known limitations [22,26]. To minimize this effect in the base-line testing, the study was started more than one year after the participants had been exposed to previous skill training.

Although personnel, equipment and lack of direct light were arranged similar to prehospital daylight conditions, one may argue that the comfort of the lab-environment may improve performance. For instance, the mental stress of an unanticipated CICV situation cannot be simulated.

Another limitation may be the homogeneity of the population of participants of the study. The results may not be directly applicable to services and clinical situations in which other types of health care providers perform cricothyrotomy.

## Conclusions

Testing the base-line PCK skills of prehospital anaesthesiologists revealed low confidence, sub-optimal performance, a failure rate of 40% and a high tracheal injury rate of 60%. The BACT technique at the base-line level demonstrated a significantly higher success rate (95%) and no tracheal damage.

After the intensive training package, a one hundred percent success rate was achieved with both techniques, and a reduction in time to successful insertion was found for both techniques. The mean time used to secure the porcine airway with PCK was significantly lower than for BACT and may be of clinical importance.

The clinicians rated a higher confidence in BACT in all phases of this study. The difference, however, was reduced after completion of the intensive training package. Based on our findings, it is likely that adequate PCK performance can only be achieved if intensive training is performed on a regular basis. If a medical system is not able to provide its physicians with sufficient training, BACT is the better choice.

## Competing interests

No author has any conflict of interest with regard to the material discussed in this manuscript.

## Authors’ contributions

ARN, PPB and MS participated in the design and writing of the manuscript. ARN and MS performed the data sampling, and ARN performed the statistical analysis. All authors read and approved the final manuscript.
